# Perseverance with technology-facilitated home-based upper limb practice after stroke: a systematic mixed studies review

**DOI:** 10.1186/s12984-021-00819-1

**Published:** 2021-02-24

**Authors:** Bridee A. Neibling, Sarah M. Jackson, Kathryn S. Hayward, Ruth N. Barker

**Affiliations:** 1 College of Healthcare Sciences, James Cook University, Townsville, QLD 4811 Australia; 2 Centre for Rural and Remote Health, James Cook University, Mount Isa, QLD 4825 Australia; 3 Melbourne School of Health Sciences, University of Melbourne, Melbourne, VIC 3084 Australia; 4 Stroke Theme, Florey Institute of Neuroscience and Mental Health, Melbourne, VIC 3084 Australia; 5 NHMRC CRE in Stroke Rehabilitation and Brain Recovery, Melbourne, VIC 3084 Australia; 6 College of Healthcare Sciences, James Cook University, Cairns, QLD 4878 Australia

**Keywords:** Home, Perseverance, Practice, Stroke, Technology, Upper limb

## Abstract

**Background:**

Technology is being increasingly investigated as an option to allow stroke survivors to exploit their full potential for recovery by facilitating home-based upper limb practice. This review seeks to explore the factors that influence perseverance with technology-facilitated home-based upper limb practice after stroke.

**Methods:**

A systematic mixed studies review with sequential exploratory synthesis was undertaken. Studies investigating adult stroke survivors with upper limb disability undertaking technology-facilitated home-based upper limb practice administered ≥ 3 times/week over a period of ≥ 4 weeks were included. Qualitative outcomes were stroke survivors’ and family members’ perceptions of their experience utilising technology to facilitate home-based upper limb practice. Quantitative outcomes were adherence and dropouts, as surrogate measures of perseverance. The Mixed Methods Appraisal Tool was used to assess quality of included studies.

**Results:**

Forty-two studies were included. Six studies were qualitative and of high quality; 28 studies were quantitative and eight were mixed methods studies, all moderate to low quality. A conceptual framework of perseverance with three stages was formed: (1) getting in the game; (2) sticking with it, and; (3) continuing or moving on. Conditions perceived to influence perseverance, and factors mediating these conditions were identified at each stage. Adherence with prescribed dose ranged from 13 to 140%. Participants were found to be less likely to adhere when prescribed sessions were more frequent (6–7 days/week) or of longer duration (≥ 12 weeks).

**Conclusion:**

From the mixed methods findings, we propose a framework for perseverance with technology-facilitated home-based upper limb practice. The framework offers opportunities for clinicians and researchers to design strategies targeting factors that influence perseverance with practice, in both the clinical prescription of practice and technology design. To confirm the clinical utility of this framework, further research is required to explore perseverance and the factors influencing perseverance.

*Registration:* PROSPERO CRD42017072799—https://www.crd.york.ac.uk/prospero/display_record.php?RecordID=72799

## Introduction

Upper limb (UL) recovery after stroke is a long and often arduous journey. High doses of task-specific therapy have been suggested to enhance neuroplasticity, motor relearning and recovery [1, 2]. Yet, the specific dose and timing of UL practice required to maximise functional recovery remains unclear [3]. Stroke survivors in the inpatient setting have been observed to complete on average 18 min per day of UL therapy, which is considered insufficient for functional recovery [4]. In turn, up to 65% of stroke survivors have a non-functional UL six months after stroke [5]; extending their UL recovery journey beyond the inpatient rehabilitation phase and into the home.

Upper limb home exercise programs (HEP) are commonly provided to stroke survivors in an effort to increase practice and enhance recovery [6]. Dose and content of UL HEP are variable, ranging from a structured one-size-fits-all program, to an individualised program specific to the needs and goals of the stroke survivor [6]. Adherence to HEP after stroke has been attributed to family support, confidence in therapist knowledge and experience, and goal oriented practice with an accountability strategy [7–9]. Non-adherence with HEP after stroke has been attributed to fatigue, depression and diminished motivation, musculoskeletal issues, and lack of time due to competing commitments [8, 9]. Additionally, some stroke survivors have found that traditional HEP are not enjoyable, too difficult or insufficiently challenging, and thus of minimal functional benefit [8–10]. Evidently, practicing intensely in the home over a long period of time is challenging for stroke survivors. Therefore, options that enable stroke survivors to continue with home-based practice in the long term need to be considered.

Technology offers an increasing number of options to facilitate independent, intensive and task-specific UL practice in the home [11]. Upper limb rehabilitation technology typically allows stroke survivors to play motion-based games on an interactive platform that offers feedback on performance and results [11]. Practice is monitored and progressed either in person or online by a therapist [11]. Some technologies also provide mechanical assistance to make practice possible [11]. Unfortunately, adherence with technology-facilitated practice is variable, and has been reported to be lower than that of more traditional methods due to decreased task specificity and engagement with the technology [6]. Recommendations for technology design focus on engagement, including personalisation of games and sufficient variability and challenge, as well as user-friendliness and contextual applicability to the home environment [11]. To date however, within efficacy studies of technology-facilitated interventions, there has been limited exploration of how these design factors influence stroke survivors’ ability to persevere with practice.

Perseverance is a dynamic behaviour that has been defined as “persistence in doing something despite difficulty or delay in achieving success” [12] and is known to be influenced by multiple factors[13, 14]. Perseverance is thought to play a vital role in disciplines where significant amounts of practice over years are required to achieve expert skill [14–16]. Accordingly, perseverance is required by stroke survivors to recover UL skill through high dose practice, over a long period of time, to promote the neuroplasticity required for recovery [1, 2]. The added challenge for stroke survivors is that they must persevere in the presence of physical and cognitive impairments, and independently within their home environment. While technology offers a unique opportunity to enhance independent home-based UL practice, the factors that influence stroke survivors’ ability to persevere with technology-facilitated practice are yet to be explored in detail. Therefore, the question to be answered in this systematic mixed studies review was: What are the factors that influence perseverance with technology-facilitated home-based UL practice after stroke?

## Methods

A systematic mixed studies review with sequential exploratory synthesis was conducted [17, 18]. Mixed-methods were used to gain a more thorough understanding of the complex phenomenon of perseverance, and to corroborate qualitative and quantitative findings to provide meaningful and relevant evidence to use in both the prescription and design of technology for rehabilitation [17, 18]. The review was reported according to the Preferred Reporting Items for Systematic Reviews and Meta-Analyses (PRISMA) guideline [19]. Key definitions for this review are outlined in Box 1.

Box 1. Key definitions *Perseverance*—Persistence with UL practice despite difficulty or delay in achieving success[12]

*Home*—A stroke survivor’s place of residence; specifically excluding hospitals, residential aged care facilities, assisted living units, transitional rehabilitation houses, or residences custom built to trial technology (e.g. Smart Apartments).

*Technology*—Any device incorporating a hardware system and interactive software which responds to the user's actions by presenting content such as text, moving image, animation, video, audio, and video games with the goal of promoting recovery[20]

*Dose*—Amount of practice expressed in time (minutes or hours) or repetitions/session, sessions/day, days/week, number of weeks.

*Phase of recovery*—Hyper-acute (0–24 h), acute (1–7 days), early subacute (7 days–3 months), late subacute (3–6 months), and chronic (> 6 months)[21]

### Identification and selection of studies

Studies were identified through the information sources outlined in Box 2. A search strategy combining MeSH terms and keywords was developed by the research team in consultation with an experienced librarian (Additional file 1: Medline search strategy). Database searches were performed on the 24th of March 2020.

Eligibility criteria, presented in Box 3, were defined a priori. To determine the peer review status of a journal, Ulrich’s Web (http://ulrichsweb.serialssolutions.com/) was consulted. We cross-checked all non peer-reviewed journal outcomes with the journal website to confirm exclusion. If more than one study utilised a single sample and presented the same data, the most recent study was included; however if unique data were presented, all studies were included. Two reviewers (BN, SJ) independently screened titles, abstracts, and full texts. Disagreements regarding eligibility were discussed and if not resolved were mediated by a third reviewer (RB).

Box 2. Information sources *Databases*—MEDLINE, CINAHL, PsychINFO, Scopus, Web of Science, EmCare.

*Identification of additional literature*—Hand searching of reference lists of (a) systematic reviews identified in search, (b) included articles, and (c) forward tracking of citations of included articles using Google Scholar; and through consultation with field experts.

Box 3. Eligibility criteria *Design*—Qualitative, quantitative or mixed methods research designs.

*Participants*—Stroke survivors ≥ 18 years of age with any level of UL disability.

*Intervention*—Technology used as a medium to perform home-based UL practice where the prescribed dose was ≥ 3x/week for ≥ 4 weeks^a^.

*Qualitative outcomes*—Perseverance related outcomes were drawn from perceptions of stroke survivors experience of utilising technology to facilitate home-based UL practice; reported by stroke survivors and their significant others, and therapists and researchers.

*Quantitative outcomes*—Perseverance related outcomes (e.g. Adherence, usability, satisfaction, UL outcomes).

*Publishing conditions*—Published from 2010–2020^b^ in English language in a peer reviewed journal.

^a^Authors believed that anything less than four weeks was insufficient to demonstrate perseverance. Additionally, the median length of UL rehabilitation trials is 3.5 weeks therefore choosing a lower limit higher that is than four weeks would exclude many trials [22].

^b^Ten years was chosen as the search window to correspond with the period of technological advances in therapeutic devices for UL rehabilitation.

### Assessment of study quality

The Mixed Methods Appraisal Tool (MMAT) was used to assess quality of the included articles [23]. The MMAT is a critical appraisal tool designed for use in systematic mixed studies reviews [23]. It can be used to assess qualitative studies, randomised controlled trials, non-randomised trials, quantitative descriptive studies and mixed methods studies [23]. Two researchers (BN, SJ) independently assessed each study. Studies that satisfied the MMAT criteria scored a “Y”, whereas studies that either did not satisfy the criteria, or provided insufficient information to adequately assess the criteria, scored an “N” [23]. Disagreements in scores were discussed and if not resolved were mediated by a third reviewer (RB).

### Data extraction, analysis, and synthesis

A sequential exploratory synthesis was conducted [17, 18]. Sequential exploratory synthesis involves an initial qualitative data collection and analysis phase which subsequently informs, and is integrated with quantitative data collection and analysis to produce overall interpretations [17, 18]. Qualitative data were extracted by one reviewer (BN) and managed using NVivo^1^ software. Data extracted consisted of terminology and concepts relating to perseverance, contained in reports from stroke survivors and their significant others, and therapists and researchers, regarding stroke survivors’ experience of utilising technology to facilitate home-based UL practice. Qualitative data were analysed thematically in a stepwise process [24]. Firstly the primary reviewer (BN) familiarised herself with the data by reading the studies in full, and documenting thoughts about potential factors influencing perseverance. Factors were then coded and collated into overarching stages of perseverance (BN). The factors and stages were refined through 5–10 iterative cycles of mind mapping to make sense of the connections between themes (BN), returning to the raw data to ensure referential adequacy (BN), and discussions to vet and confirm consensus on final factors and stages (BN, SJ, RB, KH) [24].

Qualitative findings of factors perceived to influence perseverance drove the extraction of quantitative data pertaining to study characteristics, intervention characteristics, and perseverance-related outcomes (Box 4). Data extracted were entered into Microsoft Excel^2^ by one reviewer (BN), and confirmed by a second reviewer (SJ). Perseverance-related outcomes were used in the absence of an established measure of perseverance. Quantitative data was analysed descriptively by one reviewer (BN). To explore a clinically important difference statistically and clinically significant results within the experimental group were reported. Clinical significance was recorded for UL outcomes if the difference between pre and post intervention score exceeded the minimal clinically important difference (MCID) for the outcome measure of interest [25]. Two researchers (BN, RB) cross referenced qualitative themes and quantitative data to identify areas of convergence and divergence [17, 18].

Box 4. Quantitative data *Study characteristics*—number of participants, gender ratio, stroke chronicity, and dose.

*Intervention characteristics*—type of intervention, hardware and software, commercial availability, rehabilitation specificity, set-up and training assistance required, mechanical device assistance, feedback provided, and progression of training.

*Surrogate measurement of perseverance—*

- *Adherence*—expressed as a percentage of *prescribed* dose, according to average dose *achieved* and *reported* hierarchically across repetitions or time on task, time in therapy, number of sessions, or general adherence report.^a, b, c^
- *Dropouts*—Participants enrolled in the study and commenced the intervention but did not complete the intervention and/or immediate post intervention follow-up measures

*Measurement of factors perceived to influence perseverance—*

- *Usability*—System Usability Scale [26]
- *Satisfaction*—Participant Satisfaction Survey
- *Motivation*—Intrinsic Motivation Inventory [27]
- *UL Outcomes*—UL impairment, activity or participation outcomes

^a^Average dose was used for all calculations except where dose information was either not available or average dose could not be calculated from the available data

^b^The hierarchy reflects practice achieved based on most- to least-accurate quantification of dose [3]: 1. Repetitions or time on task; 2. Time in therapy; 3. Number of sessions; and 4. General adherence report (e.g. all participants completed the protocol).

^c^Experimental group only

## Results

### Flow of studies through the review

**Fig. 1:**
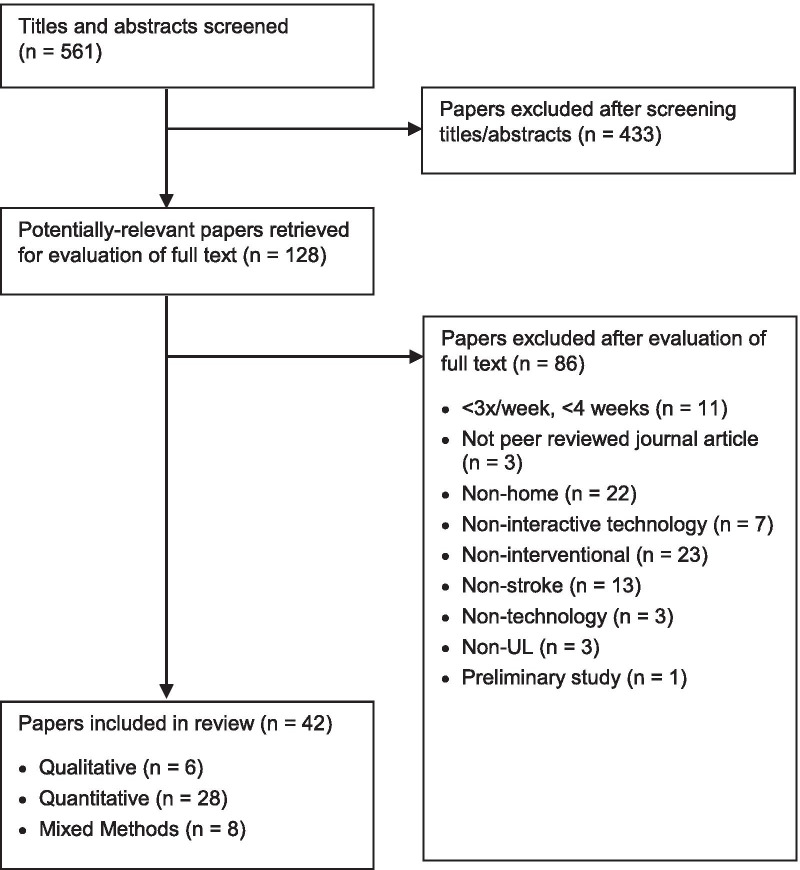
Flow of studies through the review

A total of 1450 articles were identified. Following removal of duplicates, 561 titles and abstracts were screened for eligibility. From 128 full texts, 42 studies were included [10, 28–68]: six qualitative [10, 39, 52, 54, 59, 65], 28 quantitative [28, 30, 31, 33–38, 40, 43–51, 53, 57, 58, 60, 62, 64, 66–68], and eight mixed methods [29, 32, 41, 42, 55, 56, 61, 63]. Figure 1 presents the flow of studies and reasons for exclusion.

### Characteristics of studies

Study characteristics are summarised in Table 1 [10, 28–68]. Overall, 692 stroke survivors participated in UL interventions across 42 studies [10, 28–68]. There were 10 instances where data from one sample was reported in two included studies [10, 28–30, 36–39, 49, 50, 52, 55, 57–59, 61, 62, 65, 67, 68]. Where duplication of the sample and intervention received existed, participants were counted once. There was a total of 407 male and 265 female stroke survivors from 39 studies that reported gender [10, 28–31, 33–39, 41–43, 45, 47–68]. Average age of stroke survivors ranged from 49 to 83 years [10, 28–68]. Eighty-eight percent of studies included participants who were stroke survivors in the *chronic* phase of recovery [10, 28–31, 33–39, 41–47, 50–66, 68]. Upper limb function was mixed, however classification by severity of UL disability was not possible due to the variance of disability measures employed and lack of a gold standard classification measure (Additional file 2: Characteristics of upper limb disability) [10, 28–68]. Twenty-five family members/caregivers, and seven therapists contributed to qualitative data, with one study not reporting the number of caregivers involved [35, 39, 42, 52, 54, 59, 65].

**Table 1:** Characteristics of studies and outcomes

Author (Year) Country	Study design	Participants	Intervention	Qualitative perseverance data collection and outcomes				Quantitative perseverance data collection and outcomes					
				Data collection method	Stage of perseverance			Surrogate measures	Outcomes for surrogate measures	Measures of factors	Outcomes for measures of factors		
					1	2	3				SS	CS	Other
*Qualitative*													
Donoso-Brown (2015) USA [10]	Qualitative descriptive	n = 10^a^ 10 stroke survivors 56.3 (44–69) years 6M:4F Chronic	NGT 45 min/session 1 session/day 5 days/week 4 weeks	Semi-structured interviews	X	X	X	–	–	–	–	–	
Emmerson (2018) Australia [39]	Phenomenology	n = 10^b^ 10 stroke survivors 2 family members 72 (51–85) years 10M:0F Early Subacute-Chronic	iPad Dose variable 4 weeks	In-depth semi- structured interviews	X	X	X	–	–	–	–	–	
O’Brien Cherry (2017) USA [52]	Ethnography	n = 10^c^ 10 stroke survivors Unknown number of family members 62.2 (49–88) years 10M:0F Early Subacute-Chronic	HandMentor Pro™ 1-2hrs/day ≥ 1 session/day 7 days/week 12 weeks	Observation, in-depth semi- structured interviews	X	X	X	–	–	–	–	–	
Parker (2014) UK [54]	Realistic evaluation	n = 5 5 stroke survivors 5 family members 71 (62–79) years 4M:1F Late Subacute-Chronic	SMART System Sessions/day not reported Days/week not reported 5 weeks	Observation, interviews, diary	X	X		–	–	–	–	–	
Sivan (2016) UK [59]	Specific qualitative methodology not reported	n = 17^d^ 17 stroke survivors 7 therapists 56.4 years 14M:3F Chronic	hCAAR 30 min/session 1 session/day 5 days/week 8 weeks	Semi- structured interviews	X	X	X	–	–	–	–	–	
Wingham (2015) UK [65]	Specific qualitative methodology not reported	n = 18^e^ 18 stroke survivors 10 family members 65 (35–84) years 11M:7F stroke survivors 8M:2F family members Early Subacute-Chronic	Nintendo Wii Sports™ 45 min/session 1 session/day 7 days/week 6 weeks	Semi- structured interviews	X	X	X	–	–	–	–	–	
*Quantitative*													
Adie (2017) UK [28]	RCT	n = 235 (E 117, C 118)^e^ E 66.8 (SD 14.6), C 68.0 (SD 11.9) years E 66M:51F, C 65M:53F Acute-Chronic	Nintendo Wii Sports™ 45 min/session 1 session/day 7 days/week 6 weeks	–	X	X		Adherence Dropouts	82%^T^ Desire not to continue (3); Medically deteriorated (4); Unable to contact (4) Incomplete data (4)	ARAT COPM EQ-5D-3L MAL SIS	NT NT NT NT NT	Y Y N U U	
Basteris (2015) Netherlands, Italy [30]	Case Series	n = 7^f^ 53.7 (34–66) years 5M:2F Chronic	SCRIPT* 30 min/session 1 session/day 6 days/week 6 weeks	–		X		Adherence	50%^T^	–	–	–	
Bernocchi (2018) Italy [31]	Case Series	n = 21 69 (62–75) years 14M:7F Early Subacute- Chronic	Glorhea Lite™ 45 min/session 1 session/day 6 days/week 8 weeks	–	X	X		Adherence Dropouts	79%^T^ Death (1) Medically deteriorated (1); Nursing home admission (1); Desire not to continue (1)	BI Grip strength mAS Motricity Index NHPT Pain VAS	Y Y N Y Y N	U U U U U U	
Brokaw (2015) USA [33]	Case Study	n = 1 49 years F Chronic	HAMSTER, HandSOME 30 min/session 1 session/day 5 days/week 4 weeks	–	X			Adherence	95%^S^	FMA JHFT SIS-16	NT NT NT	N U N	
Buick (2016) Canada, Ireland [34]	Case Series	n = 11 64.18 (54–86) years 10M:1F Chronic	ReJoyce and FES-ET 60 min/session 1 session/day 5 days/week 6 weeks	–		X		Dropouts	Failed to complete intervention (1)	ARAT	Y	N	
										PIADS	–	–	Moderate positive impact on measures of competence (+ 1.01) adaptability (+ 1.1) Slight positive impact on self-esteem (+ 0.46)
Burdea (2019) USA [35]	Case Series	n = 7 7 stroke survivors 7 caregivers 64.14 (48–79) stroke survivors 60.29 (27–74) caregivers 4M:3F stroke survivors 5M:2F caregivers Chronic	BrightBrainer 20–40 min/ session 1 session/day 5 days/week 4 weeks	–	X		X	Adherence	105%^T^	CAHAI	Y	N	
										Caregiver feedback survey	–	–	Nil scores > 4/5
										FMA	N	N	
										Grip strength	Y	U	
										JHFT	N	U	
										Pinch strength	N	U	
										Stroke survivor feedback survey	–	–	Score > 4/5 for: useful instruction, decreased boredom, encouraging others to use the device, liking the system overall
										UEFI	N	Y	
										UL ROM	N	U	
Butler (2014) USA [36]	Case Series	n = 13 (UL 9)^c^ 59.7 (41–74) years 12M:1F Early Subacute-Chronic	HandMentor Pro™ 1–2hrs/session ≥ 1 session/day 7 days/week 12 weeks	–	X	X	X	Adherence Dropouts	13%^T^ Unknown (1)	ARAT	Y	Y	
										FIM-C	N	N	
										FIM-M	Y	N	
										Satisfaction Survey	–	–	‘high’ satisfaction
Donoso-Brown (2014) USA [37]	Interrupted time series with non- parallel control	n = 12^a^ 60 (47–69) years 5M:4F Chronic	NGT 45min/session 1 session/day 5 days/week 4 weeks	–		X	X	Adherence Dropouts	79%^T^ Medically deteriorated (1); Lack of time (2)	CAHAI	N	N	
										ROM	N	U	
										sEMG-Game Play	N	U	
										sEMG-Lab Assessment	Y	U	
										WMFT	N	N	
Emmerson (2017) Australia [38]	RCT	n = 62 (E 30, C 32)^b^ E 68 (SD 15), C 63 (SD 18) years E 17M:13F, C 19M:13F Early Subacute-Chronic	iPad Dose variable 4 weeks	–			X	Adherence Dropouts	62%^G^ Death (1); Good progress (3)	Grip Strength	NT	N	
										Satisfaction Survey	–	–	E 71%, C 67%
										WMFT-Quality	NT	N	
										WMFT-Time	NT	N	
Fluet (2019) USA [40]	RCT	n = 11 (E 5, C 6) E 58 (SD9), C 65 (SD 15) years M:F not reported Time since stroke not reported	HoVRS* 20 min/session ≥ 1 session/day 7 days/week 12 weeks	–		X		Adherence	68%^T^	BBT	NT	U	
										FMA	NT	U	
										IMI	–	–	E participants (n = 5) scored higher than C participants (n = 3) at pre- and post-intervention overall, and on interest/ enjoyment subscales
Housley (2016) USA [43]	Case Series	n = 20 (UL 10) 67(± 11.4) years 19M:1F Early Subacute-Chronic	HandMentor Pro™ 1-2hrs/session ≥ 1 session/day 7 days/week 12 weeks	–	X	X	X	Adherence Dropouts	79%^T^ Medically deteriorated (1)	ARAT	Y	Y	
										FIM-Total	N	N	
										Satisfaction Survey	–	–	‘high’ satisfaction
Jordan (2014) NZ [44]	Interrupted time series with non- parallel control	n = 13 68.6 (56.3–79.6) years M:F not reported Chronic	Smart Skate 45–60 min/ session 1 session/day 3–5 days/week 4–6 weeks	–	X			Dropouts	Personal reasons (1)	FMA	Y	N	
										IMI	–	–	Average 6.12/7
										Strength	Y	U	
King (2012) NZ [45]	Case Series	n = 3 56.3 (47–65) years 2M:1F Chronic	CyWee Z Participant selected dose 8 weeks	–	X	X		Adherence	100%^G^	DASH	NT	U	
										FMA	NT	N	
										IMI	–	–	Interest/ enjoyment: 31.33/49 Perceived choice: 18.33/49 Perceived competence: 28/42 Value/usefulness: 43.33/49 Effort/importance: 20.66/35
Langan (2013) USA [46]	Case Series	n = 7 56 (47–63) years 5M:2F Chronic	Stereognosis Training System 60 min/session 1 session/day 5 days/week 6 weeks	–		X		Adherence Dropouts	90%^G^ Unknown (1)	Kinematics	Y	U	
										Proprioception – Joint Movement	N	U	
										Proprioception – Tactile Discrimination	N	U	
										WMFT	NT	U	
Lin (2013) USA [47]	Case Series	n = 2 52.5 (48–57) years 0M:2F Chronic	Looking Glass 60 min/session 1 session/day 7 days/week	–	X	X		Adherence	71%^S^	ARAT COPM MAL ROM	NT NT NT NT	Y Y U U	
Linder (2013) USA [48]	Case Study	n = 1 54 years M Late Subacute	HandMentor Pro™ 3 hrs/day (2hours HMP, 1hour HEP) 5 days/week 8 weeks	–	X	X	X	Adherence	56%^T^	ARAT	NT	Y	
										FMA	NT	Y	
										SIS-ADL	NT	Y	
										SIS-Hand Function	NT	N	
										SIS-Mobility	NT	Y	
										SIS-Strength	NT	N	
										WMFT-Time	NT	Y	
										WMFT- Quality	NT	Y	
Linder (2015) USA [49]	RCT	n = 99 (E 51, C 48)^g^ E 59.4(SD 13.6), C 55.5 (SD 12.6) years E 31M:20F, C 33M:15F Late Subacute	HandMentor Pro™ 3 hrs/day (2hours HMP, 1hour HEP) 5 days/week 8 weeks	–	X	X		Adherence Dropouts	112%^T^ Unknown (8)	SIS-ADL	Y	Y	
										SIS-Hand Function	Y	Y	
										SIS-Mobility	Y	Y	
										SIS-Strength	Y	Y	
Nijenhuis (2015) Netherlands, Italy, UK [50]	Case Series	n = 24^f^ 59 (34–80) years 10M:11F Chronic	SCRIPT* 30 min/session 1 session/day 6 days/week 6 weeks	–	X	X		Adherence Dropouts	58%^T^ Shoulder pain (1); Technical issues (1); Desire not to continue (1)	ARAT	N	N	
										FMA	Y	N	
										IMI	–	–	5.2 (SD 0.9)
										MAL	N	N	
										SIS-ADL	N	N	
										SIS-Hand Function	N	N	
										SIS-Mobility	Y	N	
										SIS-Strength	Y	Y	
										SUS	–	–	69% (SD 17)
Nijenhuis (2017) Netherlands [51]	RCT	n = 20 (E 10, C 10) E 58 (48–65), C62 (54– 70) years E 7M:2F; C 3M:7F Chronic	SCRIPT 30 min/session 1 session/day 6 days/week 6 weeks	–	X	X		Adherence Dropouts	66%^T^ Shoulder pain (1)	ARAT	N	N	
										BBT	N	N	
										FMA	N	N	
										Grip Strength	N	N	
										IMI	–	–	E 5.4, C 4.9
										MAL	N	N	
										SIS-Overall	N	N	
Pareto (2011) Sweden [53]	Case Series	n = 3 83 (78–87) years 3M:0F Late Subacute-Chronic	Curictus Immersive Workbench 20 min/session 1 session/day 7 days/week 12–21 weeks	–	X			Adherence	18%^T^	ARAT BBT EQ-5D-3L Grip Strength	NT NT NT NT	Y U Y U	
Rand (2015) Israel [57]	RCT	n = 24 (E 13, C 11)^h^ E 59.1 (33–75), C 64.9 (58–80) years E 9M:4F, C 6M:5F Chronic	Microsoft X-box Kinect™ Playstation 2 Eyetoy™ 60 min/session 1 session/day 6 days/week 5 weeks	–		X	X	Adherence Dropouts	63%^T^ Personal reasons (2); Desire not to continue (2)	ARAT BBT MAL	Y Y Y	N U N	
Sivan (2014) UK [58]	Case Series	n = 17^d^ 56.4 (SD 11.5) years 14M:3F Chronic	hCAAR 30 min/session 1 session/day 5 days/week 8 weeks	–	X	X		Adherence Dropouts	43%^T^ Inadequate space (1); Unable to use technology (1)	ABILHAND Questionnaire	Y	U	
										ARAT	Y	N	
										CAHAI	Y	Y	
										FMA	Y	N	
										mAS	Y	U	
										Reaching	Y	U	
										Strength	Y	U	
Slijper (2014) Sweden [60]	Case Series	n = 12 58 (26–66) years 5M:6F Chronic	Gaming Console Participant selected dose 5 weeks	–	X	X		Adherence Dropouts	100%^O^ Medically deteriorated (1)	ABILHAND Questionnaire	N	Y	
										ARAT	Y	Y	
										FMA	Y	Y	
										Grip Strength	Y	U	
Standen (2017) UK [62]	RCT Feasibility	n = 27 (E 17, C 10)^i^ E 59 (SD 12.03), C 63 (SD 14.06) years E 8M:9F, C 8M:2F Early Subacute-Chronic	Virtual Glove 20 min/session 3 sessions/day 7 days/week 8 weeks	–	X	X	X	Dropouts	Medically deteriorated (2); Desire not to continue (1); Family commitment (2)	MAL NEADL NHPT WMFT	Y N N N	Y U U N	
Thielbar (2020) USA [64]	Randomised Crossover Trial	n = 20 (First SU 10, First MU 10) First SU 59.7 (SD 10.5) years First MU 59.8 (SD 4.8) years First SU 8M:2F, First MU 9M:1F Chronic	VERGE 60 min/session 1 session/day 4 days/week 4 weeks	–		X		Adherence	84%^G^	FMA	Y	N	
										IMI	–	–	Value/usefulness: 6.6/7 Effort/importance: 6.4/7 Interest/enjoyment: > 5.5/7 Perceived competence: > 5.5/7
Wittmann (2016) Switzerland [66]	Case Series	n = 11 60 (42–73) years 5M:6F Late Subacute–Chronic	ArmeoSenso System Participant selected dose 6 weeks	–	X	X	X	Adherence	100%^G^	Automated Reaching Assessment	Y	U	
										FMA	Y	N	
										ROM	N	U	
										WMFT	N	Y	
Wolf (2015) USA [67]	RCT	n = 99 (E 51, C 48)^g^ E 59.1 (SD 12.2), C 54.7 (SD 14.1) years E 25M:26F, C 31M:17F Early Subacute-Late Subacute	HandMentor Pro™ 3 hrs/day (C 3 hours HEP; E 2 hours HMP, 1 hour HEP) 5 days/week 8 weeks	–	X	X		Adherence Dropouts	112%^T^ Medically deteriorated (1); Poor adherence (1); Desire not to continue (1) Moved onto other therapy (1); Incomplete data (3)	ARAT	Y	N	
										FMA	Y	Y	
										WMFT-Quality	Y	N	
										WMFT-Time	Y	N	
Yacoby (2019) Israel [68]	RCT	n = 24 (E 13, C 11)^h^ E 59.1 (33–74), C 64.9 (58–80) years E 9M:4F, C 6M:5F Chronic	Microsoft Kinect™ or Sony EyeToy™ 60 min/session 1 session/day 6 days/week 5 weeks	–	X	X	X	Adherence Dropouts	63%^T^ Personal reasons (2); Desire not to continue (2)	Perceived enjoyment	–	–	4.1/5
										Perceived exertion	–	–	11.8/20
										Perceived UL improvement	–	–	5/13 participants
										Satisfaction	–	–	3.9/5
*Mixed methods*													
Alankus (2010) USA [29]	QL: Case Study QT: Case Study	n = 1^j^ 62 years F Chronic	Looking Glass 60–75 min/ session ≥ 1 session/day 5 days/week 6 weeks	Semi- structured in-depth interviews	X		X	Adherence	101%^T^	ARAT	NT	N	
										RPS	NT	U	
Bhattacharjya (2019) USA [32]	QL: Specific qualitative methodology not reported QT: Case Series	n = 2 Age not reported M:F not reported Time since stroke not reported	mRehab* 120 reps ≥ 1 session/day 5 days/week 6 weeks	Interview	X	X		Adherence	75%^S^	–	–	–	
Fu (2019) USA [41]	QT: Case Series QL: Specific qualitative methodology not reported	n = 7 59.42 (40–77) years 4M:3F Chronic	CCFES 45 min/session 2 sessions/day 6 days/week 12 weeks	Participant journals Interview	X	X	X	Adherence Dropouts	110%^T^ Poor adherence (1); Medically deteriorated (1); Other commitment (2)	AMAT FMA	NT NT	U N	
Hayward (2015) Australia [42]	QL: Specific qualitative methodology not reported QT: Case Study	n = 1 1 stroke survivor 1 caregiver 57 years M Chronic	SMART Arm 60–80 reps/session ≥ 1 session/day 5 days/week 4 weeks	Observation, researcher and participant journals	X	X	X	Adherence	140%^R^	MAL	NT	Y	
										mAS	NT	U	
										MAS	NT	U	
										Ritchie Articular Index	NT	U	
										SIS	NT	U	
										Strength	NT	U	
										Tardieu Scale	NT	U	
Proffitt (2011) USA [55]	QL: Specific methodology not reported QT: Case Study	n = 1^j^ 62 years F Chronic	Looking Glass 60–75 min/ session 1 session/day 5 days/week 6 weeks	Observation, semi- structured interviews, participant journal		X	X	Adherence	100%^G^	ACS ARAT ROM RPS	NT NT NT NT	U N U U	
Proffitt (2015) USA [56]	QL: Grounded Theory QT: Case Series	n = 4 57.25 (54–64) years 2M:2F Chronic	Mystic Isle 4 hrs/week 6 weeks	Semi- structured interviews		X		Adherence	63%^T^	COPM	NT	N	
										FMA	NT	N	
										SS-QOL	NT	U	
										SUS	–	–	2.70/5
Standen (2015) UK [61]	QL: Specific methodology not reported QT: Prospective Cohort Study	n = 13^i^ 59 (40–82) years 5M:8F Early Subacute-Chronic	Virtual Glove 20 min/session 3 sessions/day 7 days/week 8 weeks	Interviews	X	X	X	Adherence Dropouts	17%^T^ Medically deteriorated (2); Family commitment (2)	–	–	–	
Szturm (2020) Canada [63]	QL: Qualitative Descriptive QT: Case Series	n = 10 58 (SD 12) years 6M:4F Late Subacute-Chronic	GTP 20 min/session 1 session/day 4 sessions/week 16 weeks	Semi- structured interviews	X	X		Adherence Dropouts	100%^T^ Unable to use technology (2)	Grip Strength	NT	N	
										Movement onset time – movement variance	NT	U	
										Object manipulation – average	NT	U	
										Object manipulation – success rate	NT	U	
										WMFT – Quality	NT	Y	
										WMFT – Time	NT	Y	

“Outcomes for measures of factors” within experimental group

*Participants self-selected dose; ^a−j^Sample duplication; Adherence based on: ^G^General adherence reported; ^R^reps/time on task; ^S^number of sessions; ^T^time in therapy

*ACS* Activity Card Sort, Acute—1–7 days post stroke, *AMES* Assisted Movement With Enhanced Sensation, *ARAT *Action Research Arm Test, *BBT* Box and Block Test, *BI *Barthel Index, *C* Control, *CAHAI* Chedoke Arm and Hand Activity Inventory, *CCFES *Contralaterally Controlled Functional Electrical Stimulation, *Chronic*  > 6 months post-stroke, *COPM* Canadian Occupational Performance Measure, *CS *Clinically Significant Change, *DASH* Disabilities of the Arm, Shoulder and Hand, *E* Experimental, *Early Subacute* 7 days–3 months post-stroke, *EQ-5D-3L* EuroQual Health State Record, *F* Female, *FIM* Functional Independence Measure, *FMA* Fugl-Meyer Assessment, *hCAAR* Home-based Computer Assisted Arm Rehabilitation, *GTP* Game-assisted Telerehabilitation Platform, *HAMSTER* Home Arm Movement Stroke Training Environment, *HandSOME* Hand Spring Operated Movement Enhancer, *Hyper-acute* 0-24hours post-stroke, *IMI* Intrinsic Motivation Inventory, *JHFT* Jebsen Hand Function Test, *Late Subacute* 3-6 months post-stroke, *M* Male, *MAL* Motor Activity Log, *mAS* Modified Ashworth Scale, *MAS* –Motor Assessment Scale, *mRehab* Mobile Rehabilitation, *MU* Multi-user, *N* No, *NEADL* Nottingham Extended Activities of Daily Living Scale, *NGT* NeuroGame Therapy, *NHPT* Nine Hole Peg Test, *NT* Statistical Significance Not Tested, *P* Participant, PIADS Psychosocial Impact of Assistive Devices Scale, *QL* Qualitative, *QT* Quantitative, *ReJoyce* The Rehabilitation Joystick for Arm and Hand Exercises, *RCT* Randomised Controlled Trial, *ROM* Range of Motion, *RPS* Reaching Performance Scale, *SCRIPT* Supervised Care and Rehabilitation Involving Personal Telerobotics, *SD* Standard Deviation;. *sEMG* Surface Electromyography, *SIS* Stroke Impact Scale, *SMART Arm* Sensorimotor Active Rehabilitation Training of the Arm, *SS* Statistically Significant Change, *SS-QOL* Stroke Specific Quality of Life Scale, *SU* Single-user, SUS System Usability Scale, *U *unable to determine CS (no MCID, insufficient data), *VERGE* Virtual Environment for Rehabilitative Gaming Exercises, WMFT Wolf Motor Function Test, *Y* Yes

Six qualitative and eight mixed-methods studies used interviews [10, 29, 32, 39, 41, 52, 54–56, 59, 61, 63, 65], observation [42, 52, 54, 55], and participant/researcher journals [41, 42, 54, 55] to collect data from stroke survivors, carers and significant others, and therapists about their experiences of using technology to facilitate home-based UL practice. No quantitative or mixed-methods studies collected direct measures of perseverance. Surrogate measures of perseverance were reported for all quantitative (Adherence [28, 30, 31, 33, 35–38, 40, 43, 45–51, 53, 57, 58, 60, 64, 66–68], Dropouts [28, 31, 34, 36–38, 43, 44, 46, 49–51, 57, 58, 60, 62, 67, 68]) and mixed-methods studies (Adherence [29, 32, 41, 42, 55, 56, 61, 63], Dropouts [41, 61, 63]). Factors perceived to influence perseverance were measured in 27 quantitative studies (Usability [50], Satisfaction [35, 36, 38, 43, 68], Motivation [34, 40, 44, 45, 50, 51, 64], UL Outcomes [28, 31, 33–38, 40, 43–51, 53, 57, 58, 60, 62, 64, 66–68]) and six mixed methods studies (Usability [56], UL Outcomes [29, 41, 42, 55, 56, 63]).

### Study quality

A detailed breakdown of quality ratings according to the MMAT are displayed in Table 2 [23]. The six qualitative studies were of high quality [10, 39, 52, 54, 59, 65], while the 28 quantitative [28, 30, 31, 33–38, 40, 43–51, 53, 57, 58, 60, 62, 64, 66–68] and eight mixed methods [29, 32, 41, 42, 55, 56, 61, 63] studies were of moderate to low quality [23]. No studies were excluded based on quality.

**Table 2:** Quality appraisal

Study	MMAT quality assessment																					
	Screening		Qualitative					Quantitative RCT					Quantitative NRT					Mixed methods				
	S1	S2	1.1	1.2	1.3	1.4	1.5	2.1	2.2	2.3	2.4	2.5	3.1	3.2	3.3	3.4	3.5	5.1	5.2	5.3	5.4	5.5
Adie (2017) [28]	Y	Y						Y	Y	Y	N	N										
Alankus (2010) [29]	Y	Y	Y	Y	N	Y	N						Y	Y	Y	Y	Y	Y	N	N	N	N
Basteris (2015) [30]	Y	Y											Y	Y	Y	N	Y					
Bernocchi (2018) [31]	Y	Y											Y	Y	N	N	Y					
Bhattacharjya (2019) [32]	Y	Y	N	N	N	N	N						N	Y	Y	Y	Y	N	N	N	N	N
Brokaw (2015) [33]	Y	Y											N	Y	Y	N	Y					
Buick (2016) [34]	Y	Y											Y	Y	Y	Y	Y					
Burdea (2019) [35]	Y	Y											Y	Y	Y	Y	Y					
Butler (2014) [36]	Y	Y											N	Y	Y	N	Y					
Donoso-Brown (2014) [37]	Y	Y											Y	Y	N	N	Y					
Donoso-Brown (2015) [10]	Y	Y	Y	Y	Y	Y	Y															
Emmerson (2017) [38]	Y	Y						Y	N	Y	Y	N										
Emmerson (2018) [39]	Y	Y	Y	Y	Y	Y	Y															
Fluet (2019) [40]	Y	Y						N	N	Y	N	N										
Fu (2019) [41]	Y	Y	N	N	N	Y	N						Y	Y	N	Y	N	N	Y	Y	N	N
Hayward (2015) [42]	Y	Y	N	Y	Y	N	Y						Y	Y	Y	N	Y	N	Y	Y	N	Y
Housley (2016) [43]	Y	Y											N	Y	Y	N	Y					
Jordan (2014) [44]	Y	Y											Y	Y	Y	N	N					
King (2012) [45]	Y	Y											Y	Y	Y	N	N					
Langan (2013) [46]	Y	Y											N	Y	Y	N	Y					
Lin (2013) [47]	Y	Y											Y	Y	Y	N	Y					
Linder (2013) [48]	Y	Y											N	Y	Y	N	Y					
Linder (2015) [49]	Y	Y						N	Y	Y	N	Y										
Nijenhuis (2015) [50]	Y	Y											Y	Y	Y	N	Y					
Nijenhuis (2017) [51]	Y	Y						N	Y	Y	N	N										
O-Brien Cherry (2017) [52]	Y	Y	Y	Y	Y	Y	Y															
Pareto (2011) [53]	N	Y											Y	Y	Y	N	Y					
Parker (2014) [54]	Y	Y	Y	N	N	Y	Y															
Proffitt (2011) [55]	Y	Y	N	Y	N	Y	N						N	Y	Y	Y	Y	N	Y	N	Y	N
Proffitt (2015) [56]	Y	Y	N	N	N	Y	N						Y	Y	N	Y	Y	Y	Y	Y	Y	N
Rand (2015) [57]	Y	Y						N	N	Y	Y	N										
Sivan (2014) [58]	Y	Y											Y	Y	Y	N	Y					
Sivan (2016) [59]	Y	Y	N	Y	Y	Y	Y															
Slijper (2014) [60]	Y	Y											Y	Y	Y	Y	Y					
Standen (2015) [61]	Y	Y	N	Y	Y	Y	Y						Y	Y	Y	Y	Y	N	Y	Y	N	Y
Standen (2017) [62]	Y	Y						Y	N	Y	N	N										
Szturm (2020) [63]	Y	Y	Y	Y	Y	Y	Y						Y	Y	N	N	Y	N	N	N	N	Y
Thielbar (2020) [64]	Y	Y						N	Y	Y	N	Y										
Wingham (2015) [65]	Y	Y	N	Y	Y	Y	Y															
Wittmann (2016) [66]	Y	Y											Y	Y	Y	N	Y					
Wolf (2015) [67]	Y	Y						N	Y	Y	Y	Y										
Yacoby (2019) [68]	Y	Y						N	N	Y	Y	N										

*MMAT* Mixed Methods Appraisal Tool [*RCT* Randomised Controlled Trial, *NRT* Non-randomised Trial, *S1*—Are there clear research questions? *S2*—Do the collected data allow to address the research questions? 1.1—Is the qualitative approach appropriate to answer the research question? 1.2—Are the qualitative data collection methods adequate to address the research question? 1.3—Are the findings adequately derived from the data? 1.4—Is the interpretation of results sufficiently substantiated by data? 1.5—Is there coherence between qualitative data sources, collection, analysis and interpretation? 3.1—Are the participants representative of the target population? 3.2—Are measurements appropriate regarding both the outcome and exposure/intervention? 3.3—Are there complete outcome data? 3.4—Are the confounders accounted for in the design and analysis? 3.5—During the study period, is the intervention/exposure administered as intended? 5.1—Is there an adequate rationale for using a mixed methods design to address the research question? 5.2—Are the different components of the study effectively integrated to answer the research question? 5.3—Are the results adequately brought together into overall interpretations? 5.4—Are divergences and inconsistencies between quantitative and qualitative results adequately addressed? 5.5—Do the different components of the study adhere to the quality criteria of each tradition of the methods involved?], *Y* Criteria satisfied, *N* Criteria not satisfied

### Characteristics of interventions

Twenty-six separate technologies were investigated (Additional file 3: Characteristics of interventions) [10, 28–68]. Wired or wireless sensors were used to monitor UL movement in all technologies; except two studies that used an iPad [38, 39]. Interactive gaming software was used in 85% of included studies [10, 28–31, 33–37, 40, 41, 43–45, 47–53, 55–59, 63–68]. Ten technologies were commercially available [28, 31, 34, 36, 38, 39, 43–45, 47–49, 52, 55–57, 65, 67, 68], seven of which had hardware and software specifically designed for rehabilitation [31, 34, 36, 43–45, 47–49, 52, 55, 56, 67].

Level of assistance required for participant set-up was reported in 28 out of 42 studies.[10, 29, 31–34, 36–45, 49, 52, 53, 56–59, 61, 62, 65, 67, 68] Five studies reported entirely independent set-up by stroke survivors [32–34, 40, 45] and the remaining 23 studies reported set-up assistance or supervision from a carer/family member or therapist.[10, 29, 31, 36–39, 41–44, 49, 52, 53, 56–59, 61, 62, 65, 67, 68] Ten technologies offered participants partial to full assistance with UL movements during practice.[30, 31, 33, 35, 36, 40–44, 48–52, 58, 59, 67]

Audio-visual instructions or cues were provided to stroke survivors within all technologies studied [10, 28–68]. Twenty-four technologies provided audio-visual performance-based feedback [10, 28–37, 40–45, 47–68]; two exceptions were iPad and stereognosis training system [38, 39, 46]. In 64% of studies, participants received at least once weekly contact from a therapist to provide feedback, monitor performance, and progress training [10, 28–30, 33, 34, 36, 37, 41–44, 46, 48–51, 53, 55, 57, 60–63, 65, 67, 68]. Contact occurred either in-person at the participant’s home or at a clinic (33%) [29, 41, 44, 50, 51, 55, 60–62], via telephone or videoconference (44%) [10, 28, 34, 36, 37, 43, 46, 48, 49, 53, 65, 67], or a combination of the two (22%) [30, 33, 42, 57, 63, 68]. Forty-six percent of the devices used a combination of automatic performance-based training progressions and manual progressions [10, 28, 30, 31, 34, 36, 37, 42–44, 48–52, 56, 58, 59, 61–63, 65, 67].

Prescribed dose of practice varied significantly between studies in terms of parameters used and magnitude (Table 1). Participants were asked to complete between 9.5 and 161 h of practice over four to 24 weeks [10, 28–31, 33–37, 40, 41, 43–53, 55–68]. Two studies prescribed dose in repetitions, recommending 1500 repetitions over 4 weeks [42], and 3900 repetitions over 6 weeks [32]. Participants self-selected their dose in seven studies [30, 32, 40, 45, 50, 60, 66]. Adherence with the prescribed dose was ≤ 50% in five studies [30, 36, 53, 58, 61], 51% to 74% in nine studies [38, 40, 47, 48, 50, 51, 56, 57, 68], 75% to 100% in 13 studies [28, 31–33, 37, 43, 45, 46, 55, 60, 63, 64, 66], ≥ 101% in six studies [29, 35, 41, 42, 49, 67], and unreported in nine studies [10, 34, 39, 44, 52, 54, 59, 62, 65]. As duration of trials and frequency of sessions increased, adherence decreased (71% of four week trials [33, 35, 37, 42, 64] vs. 50% of 12 week trials had ≥ 75% adherence [41, 43]; 71% of training 3–5 days/week trials [29, 32, 33, 35, 37, 42, 46, 49, 55, 63, 64, 67] vs. 33% of training 6–7 days/week trials had ≥ 75% adherence [28, 31, 41, 43]). When allowed to self-select their dose, stroke survivors trained for approximately 24 min/day, 4–5 days/week [30, 32, 40, 45, 50, 60, 66].

### Sequential exploratory synthesis

**Fig. 2:**
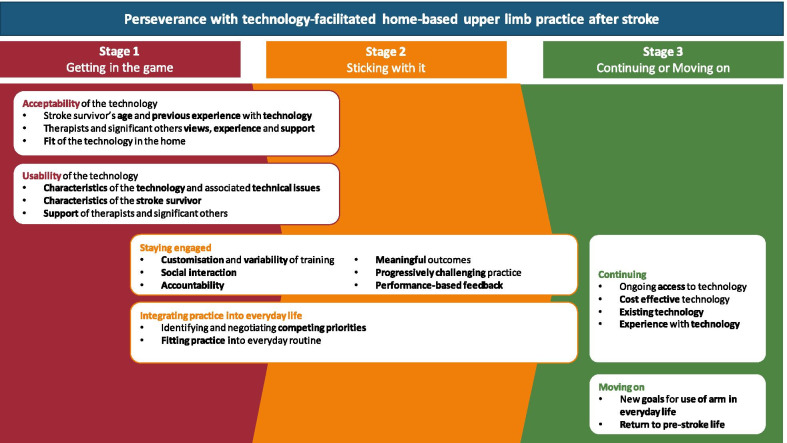
Conceptual framework for perseverance with technology-facilitated home-based upper limb practice after stroke

Perseverance with technology-facilitated practice in the home, as reported in the literature, was organised into a conceptual framework (Fig. 2). Three stages are presented on a linear continuum: (1) getting in the game; (2) sticking with it; and (3) continuing or moving on. The conditions and mediating factors perceived to influence stroke survivors’ ability to persevere with practice are organised within the stage where they appear to exert the most influence. However, factors can be fluid across the stages as demonstrated in Fig. 2. Articles contributing to thematic analysis are presented in Table 1 and supporting quotes are presented in Table 3.[10, 28–68

]

**Table 3:** Supporting quotes for thematic analysis

**Stage**	Supporting quotes
*Condition*	
Mediating factor	
**Getting in the game**	
*Acceptability*	
Age	“age of our participants was young in comparison with the population’s studies of stroke; this may have improved acceptability of the study intervention, evidenced by the low number of participants discontinuing the intervention” [28]
	“In fact, the two oldest participants were also those who had never used a computer, and also the ones who were least accepting of the technology” [39]
Previous experience	“Despite some initial apprehension and preconceived ideas, following exposure and an opportunity to try the tablet, eight participants were more open to the use of this technology, and much less anxious” [39]
	“I had some initial problems with understanding the technology but once I got into routine use (of the device), it became easy to use.” [59]
	“But I think the fact that being something different and being interactive is the main motivator with it actually, than just exercising” [65]
Support	“The therapists’ attitude towards the intervention may have influenced the participants’ attitude” [28]
	“Previous experience of rehabilitation (i.e. therapist led rehabilitation to patient led rehabilitation) could influence expectations of service delivery. One participant did not believe it was his responsibility to lead his rehabilitation, “It’s not up to me to sit here and do it all myself is it…You’re the experts you should be doing it”” [54]
Fit	“Direct observations of the home environment revealed other related barriers to use based on the physical nature of the devices themselves. Many of the homes were crowded spaces with limited room, few available electrical outlets or without a table or chair at proper height. As a result, setting up and using the device was sometimes difficult.” [52]
	“One of these two participants had reassessed his home situation (in view of some relatives living in his house for holiday) and felt there was inadequate space in his house to accommodate the device for the period of the study” [59]
*Usability*	
Device characteristics	“Participants were also asked what they would change about the device, and most responses involved adding more games with greater levels of difficulty, refining the system for sending data to the secure server after use, and making the computer component of the controller device smaller and easier to handle.” [36]
	“One of the complaints about the devices was their size and weight, and the resulting difficulty of moving them around the home as a result. One participant said, ‘‘It’s so bulky and when you move it you throw it out of whack, so you have to stay in the same place and work around it’’. Most participants reported that they did not move the device from where it was originally set up.” [52]
Personal characteristics	“For Participant 3, accessing the game on the computer (e.g., finding the icon to click, following the steps to log-in) presented a barrier to play. Participant 3 was able to play the game with verbal cues when the OT was present in the home; however at other times, she did not use assistance from caregivers to load or play the game. Due to her cognitive deficits, she had difficulty processing and following directions, both written and pictorial.” [56]
	“Patients with severe impairments of arm function used the system less than those with moderate or mild impairments” [66]
Technical issues	“Comments of dissatisfaction generally focused on technical aspects of the device, such as recurrent freezing of the computer games and taking too long/failure of the device to send data to the secure server.” [36]
	“Each participant encountered at least one instance of technological malfunction that required troubleshooting.” [10]
	“Technical issues that arose due to the glove being a prototype could restrict use. P4 reported on a few occasions that these made her want to throw the computer out of the window.” [61]
Support	“Very important aspects of our study were the educational sessions before discharge home, the caregivers’ support and the availability of the physiotherapist to manage phone call contacts, home visits and videoconference sessions, where possible.” [31]
	“JT’s dependence on his wife for set-up and manual progressions meant opportunities for practice were missed.” [42]
**Sticking with it**	
*Staying engaged*	
Social interaction	“Family support was crucial: “My granddaughter used to play the Balloonpop and encouraged me. I mean, obviously she got fantastic scores that I wouldn’t be able to achieve, but I was so there, wanting to get as much as I could.... It’s good to have other people to play with you because you said, you know, that we could set her up, and we did.” (P23)” [61]
	“The phone call seemed to play a bigger role in the intervention period for those who were house-bound or spent a significant proportion of the time on their own. It provided a form of company and a link with the ‘outside world’.” [65]
Accountability	“On Day 10, when the therapist noticed that daily time of use was lower than requested of the participant, she called the participant to encourage greater compliance. Thereafter, there was a noticeable increase in daily use” [49]
	“It is easier for him to tell other people that ‘I did catch the ball and I got 3100′ or… ‘I’m better this week I got 3600′ and even though it might not make much sense to other people, they can tell it is going up and it always seems to appeal to him and he wants to do better on it each time” [54]
Meaningful outcomes	“Marie wanted to use motion games to accomplish her own goals” [29]
	“Participants reported that being both engaged and successful motivated them to continue with NGT and this appeared to reduce, although not completely eliminate, frustration” [10]
	“Functional changes observed by the participant along with the modifications made to the games motivated her to continue playing them throughout the intervention.” [47]
	“stroke survivors and their caregivers took ownership of their rehabilitation, especially where the Wii™ was perceived as improving arm function” [65]
Customisation and Variety	“Participants were also asked what they would change about the device, and most responses involved adding more games with greater levels of difficulty” [36]
	“Participants' feedback also highlighted the importance of choosing from a variety of games to engage participants.” [10]
	“Comments of dissatisfaction generally focused on limited game selection.” [43]
	“In order to improve the users’ ability to relate to the avatar, it was suggested that the graphical interface needs to be individualised to the user. The user may wish to alter the avatar image to look like them (i.e. male/ female) and therefore, make it both recognisable and easier to relate to. One user found it difficult to relate to the avatar as she thought it did not represent her, “It’s not me on the screen, that’s a man and I’m a woman!”” [54]
	“Participants indicated they liked the customized aspect of the Mystic Isle. They claimed they were inspired to play and enjoyed the games because they were tailored to help them achieve their particular goals.” [56]
Challenge	“In many cases, subjects performed within a challenging session more repetitions than in a challenging – then supporting one – i.e. they performed better for a longer time. This confirms the effect of challenge on subjects’ motivation, i.e. by challenging the subject it is possible to extend the training intensity without affecting his/her performance. The role of challenge in motivation is also confirmed by the lower number of repetitions in under-challenging sessions compared to the under-supporting ones.” [30]
	“A few, suggested that the game was not cognitively challenging and did not motivate them” [10]
	“Three participants liked the concept of the levels becoming progressively harder as that kept them interested in using the device” [59]
	“Participants wanted to exceed a previous score or ‘beat the machine’, as some participants described it, and this spurred them on. A minority of participants felt they had become obsessed in this way and played longer than the allotted 45 min per day.” [65]
Feedback	“One of Marie’s most persistent requests was for additional feedback from the games. She wanted the games to “make me feel good” by pointing out when she had increased her range of motion or completed a task more quickly.” [29]
	“Participants enjoyed watching themselves complete exercises, and used the visual and auditory feedback to identify where they needed to work harder or in a different way.” [39]
	“JT was motivated by the 4-wk time period that the SMART Arm was available, on-screen feedback and automatic progressions in response to his success, and in-home coaching sessions during which his progress was reviewed and guidance was gained to progress further.” [42]
	“In addition, one participant explained that the feedback would be more meaningful if his results were something he could relate to in everyday life like being able to play his guitar or using his hand to hold a plectrum, “The feedback would be better if I could relate it to playing my guitar or holding the plectrum because these are things I want to be able to do… goals”” [54]
	“Because the onscreen display was sometimes inaccurate, the participants (and in some instances the carers as well) became frustrated, lost patience, and trust in the feedback provided. This resulted in the participants being less willing to use the equipment and/or dismissing the feedback as an inaccurate evaluation of their performance.” [54]
*Integrating practice into everyday life*	
Competing priorities	“many people recovering from stroke experience periods of disabling fatigue that require periods of rest throughout the day: “In my first 4 months, I was really a bit tired every day.... I don’t think I'd have had the chance to do that (use the glove).”” [61]
	“Analysis of the interviews also suggested the possibility that the patients recruited were those who were more likely to be trying to return to their prestroke life, and attempts to return to work or other activities away from the home precluded the recommended level of use of the intervention.” [62]
	“Family and life role responsibilities such as taking care of children or going to work occasionally interfered with compliance.” [67]
Fitting it in	“Six out of ten participants reported including the game into their daily routine. Hannah had it setup at the office and reports, “So it was easy to form a routine about when I would do it because I have a lot of cheerleaders there.””1 [10]
	“2 h of robotic therapy per day may be perceived as excessively burdensome, especially when coupled with 1 h of HEP activities” [47]
	“Due to the convenience of being able to use the device in their homes, participants gained a sense of control over the scheduling of their therapy.” [52]
	“tailoring game sessions and exercises so that they can be completed with minimal effort and in short amounts of time is likely to facilitate adoption on an ongoing basis by stroke survivors” [56]
	“All the participants and their caregivers were unanimous in their preference for carrying out this type of rehabilitation in their homes. This was perceived to have provided a more flexible and less stressful environment, and enabled the participants to fit their rehabilitation in with their day-to-day lives and they were able to accommodate their responsibilities, social activities and other medical appointments.” [65]
**Continuing**	
	“One suggestion made by two participants was that the length of time that participants used the system should be increased. Gerald stated that he wanted “more time, uh, just, 30 days is not time enough.” Mary concurred, reporting, “Four weeks might not be enough to see results.”” [10]
	“The two participants who owned tablets reported that they were now using them more often. Four others advised that they planned on making new purchases or borrowing family member’s tablets.” [39]
	“Two participants were interested in buying the device if it were available commercially.” [59]
	“Overall system usage and the reported desire to continue training after completion of the study protocol suggest that the therapy could even be applied over longer periods.” [66]
	“And the cost and everything else. Like if you’re in business or at [son’s] age, you can use them all the time. At our age, we’ve been good for 80 years without them, I can live a bit longer without them.” [39]
	“Furthermore, although costs are minimal for direct therapy care, the cost of robotic devices has not traditionally been covered by third-party payers.” [48]
	“Patients also responded positively to the way in which rehabilitation was delivered by the home-based robotic device itself, such as having control over the timing and duration of the rehabilitation therapy sessions, and avoiding the cost and time involved with travel to do physical therapy in a clinical setting.” [52]
	“During the follow-up period, many more participants continued to self-train using the video-games compared with traditional self training. This fact is of great importance since self-training programs need to be long term and sustainable. Most participants who continued to play the video-games also demonstrated clinical meaningful UE functional improvement during the period after the study.” [57]
**Moving on**	
	“A few also reported that they were incorporating their affected upper extremity in daily activities in new ways. Michael who had more severe impairment reported that, “When one's in a grocery store, pushing a shopping cart, most people do it with both arms or both hands, and so I'm trying now to make sure I rest my affected arm on the shopping cart handle … just at least it would start to emulate or simulate a more normal approach to ADL.” [10]
	“engaging in a home program like NGT could help a person after stroke attempt to use their affected upper extremity, yet individuals likely still need assistance in identifying which activities are most appropriate for continued practice” [10]
	“I can envision discovering more things to do.” [55]
	“All five participants who noticed improved power in their arms reported that they were using the affected arm more in daily activities and there was an improvement in functional ability in everyday tasks.” [59]
	“Inevitably, as participants recovered, they wanted to return to their prestroke life, especially if they were mobile. This barrier to use included returning to work, going on holiday, driving, or hobbies: “I’ve got my allotments to do; there’s obviously going out shopping and... trying to fit round the rest of your day.”” [61]
	“The set period of time (six weeks) appeared to motivate participants to play the Wii™ regularly. Most participants viewed the Wii™ intervention as a specific stage in the rehabilitation process, and once the six weeks had finished felt it was time to move onto other activities. “Cos you (caregiver) said, ‘I’ll go & buy you one (a Wii™)’, and I said, ‘No, no, no’…It was awful in one way it was going but, in another way, because I was getting better, um, it didn’t make me go and sit in a chair …I kept trying do little bits round the house, didn’t I?”” [65]

*ADL* activities of daily living, *NGT* neurogame therapy, *OT* occupational therapist, *SMART Arm* sensorimotor active rehabilitation training of the arm, *UE* upper extremity

### Stage 1—Getting in the game

Stroke survivors’ ability to get in the game or get started with technology-facilitated home-based UL practice appears to be mediated by acceptability and usability of the technology by the stroke survivor and their significant others.

#### Acceptability

Stroke survivors’ acceptance of technology to facilitate home-based UL practice was reportedly influenced by age and previous experience with technology, support received from therapists and significant others, and fit of the technology within the home. Older stroke survivors were generally less accepting of technology and viewed it as more appropriate for the younger generation. Anxieties about learning to use or breaking new equipment decreased acceptability for some, while other novice users found the innovative and fun nature of technology appealing. While the notion that ‘gaming is fun’ increased initial acceptance of technological interventions, fun alone was not adequate to maintain perseverance.

Stroke survivors’ acceptance of technology could be positively or negatively impacted by views, experience, and support from therapists and significant others. Stroke survivors were sometimes less accepting of technology when they perceived a lack of support in transitioning from a therapist-led rehabilitation model to a technology-facilitated model. The technology needed to fit well within the home to be accepted by stroke survivors and their significant others. Fit was not simply about physical space for the technology, but also its aesthetics and suitability to the environment within which it was placed.

#### Usability

Usability of the technology was paramount for stroke survivors to be able to practice independently or in a semi-independent manner. Variables reported to influence usability included the characteristics of the technology and associated technical issues; characteristics of the stroke survivor; and support from therapists, caregivers and family members.

Stroke survivors and their family members expressed a preference for devices to be small, easy to manoeuvre, and simple to setup and operate. Sometimes technology was not suited to the practice space and needed to be moved around the home, which was often laborious and resulted in technical issues, particularly software malfunction. Stroke survivors expressed frustration and dissatisfaction when technical issues impacted their ability to practice or interact with their therapist. Assistance from family members or therapists was often required to resolve technical issues, resulting in lost practice time. In some instances, technical issues caused participants to cease the intervention altogether.

Independent setup and training were sometimes limited by motor and sensory UL impairment or by cognitive-linguistic impairment. While family members provided assistance with set-up and practice, time spent waiting for assistance decreased practice opportunities. Therapist support in implementation, training, and resolution of technical issues increased usability.

#### Supporting evidence—getting in the game

Quantitative data to support the view that getting in the game was influenced by the acceptability of the technology were limited. Two failed recruitments and one dropout were attributed to poor fit of the device in the home [42, 58]. Four dropouts were attributed to technical issues and an inability to operate the device [50, 58, 63]. Satisfaction was found to be highest when technology was perceived to be user-friendly [35, 36, 38, 43].

### Stage 2—Sticking with it

To continue recovery, stroke survivors must stick with it. Within the included studies, continued practice required technology to be engaging and its use integrated into stroke survivors’ everyday life.

#### Staying engaged

Staying engaged was purportedly mediated by factors such as social interaction, accountability, meaningful outcomes, customisation and variability of training, challenge, and feedback.

Social interaction was found to engage and motivate stroke survivors. Some stroke survivors competed against family members in dual player games, while others played at their office, cheered on by co-workers. Networked games were suggested as a way to compete against those with a similar level of disability. Geographically or socially isolated stroke survivors found that reporting their performance to the research team was a positive form of social interaction which enhanced engagement. Occasionally, stroke survivors preferred to practice in a group environment (i.e. gym or community rehabilitation facility) as they found home-based practice isolating.

Stroke survivors reported that therapist monitoring prompted adherence to the prescribed dose. Additionally, being accountable and reporting meaningful outcomes to friends and family provided positive reinforcement of stroke survivors’ achievements, increasing self-efficacy, motivation and engagement with practice. Some stroke survivors were self-accountable, creating individual goals within games and monitoring their progress to remain engaged. When improved UL function translated into increased independence with activities of daily living, stroke survivors confidence in the technology and motivation to stick with training increased.

Stroke survivors were most engaged with technology that offered multiple game choices; limited game selection was associated with boredom. Conversely, a lack of structure in technological interventions was thought to decrease adherence in some studies. Individual customisation of games that accurately reflected the stroke survivor and their interests and goals improved engagement. Stroke survivors became disengaged when games were either not cognitively or physically challenging, or so challenging that they limited success. Automatic, performance-based progression was preferred to manual progression as an adequate level of challenge was maintained, without having to wait for assistance to manually progress.

Audio-visual feedback both within and after a game or session provided positive reinforcement, increasing motivation and engagement. Feedback of results at the end of a session was appreciated, and stroke survivors suggested displaying results along a time continuum for comparison of current and previous performance. Conversely, feedback could cause dis-engagement if it was overly negative, if participants were unable to relate it back to their goals, or if they perceived it to be an inaccurate representation of their performance.

#### Integrating practice into everyday life

Included studies recognised that for stroke survivors to stick with practice, practice needed to be integrated into their everyday life. Successful integration means working around competing priorities which limit practice opportunities, and the stroke survivor fitting practice into their normal routine. Stroke survivors’ ability to integrate practice was impacted by work and family commitments, illness, hobbies, and travel. Home-based practice was considered to be convenient and flexible. Stroke survivors could fit practice into their everyday routine more easily as it involved no travel time and minimal or no cost. Stroke survivors preferred shorter, more frequent training sessions as higher doses were more difficult to fit into the day, increased fatigue, and could feel excessively burdensome.

#### Supporting evidence—sticking with it

Quantitative data to support the view that sticking with it was influenced by staying engaged and integrating practice into everyday life were limited. Twenty-three dropouts were attributed to poor adherence and competing priorities [10, 28, 30, 31, 34, 37, 41, 43, 44, 49–51, 57, 60–62, 66–68], while 16 stroke survivors decided not to continue with their respective intervention [28, 30, 31, 49, 50, 57, 61, 62, 65, 67, 68]. Clinically significant improvement in UL outcomes for at least one measure were obtained in 16 studies [28, 35, 36, 42, 43, 47–50, 53, 58, 60, 62, 63, 66, 67], nine of which had > 75% adherence [28, 35, 42, 43, 49, 60, 63, 66, 67].

### Stage 3—Continuing OR moving on

The final stage of persevering with technology-facilitated home-based UL practice appears to be continuing or moving on from technology-facilitated practice.

#### Continuing

Some participants felt they needed to have access to the technology for a longer period of time to improve their UL function. Stroke survivors were unlikely to purchase technology that they perceived to be either too costly or superfluous. Those with existing home-based technology (e.g. iPad or Wii) or previous technology experience were more willing to persevere with practice beyond the endpoint of trials.

#### Moving on

The trial endpoint was often viewed as a time to move onto other rehabilitation activities. Participants reported setting new goals and trying to integrate use of their arm and hand into everyday activities to aid recovery. Sometimes after a trial, stroke survivors’ UL function had improved sufficiently to return to their pre-stroke life, which took precedence over continued practice with technology.

#### Supporting evidence—continuing or moving on

Quantitative data to support the view that continuing with practice was influenced by continued access to technology was limited. More stroke survivors chose to continue with technology-facilitated practice over a traditional HEP when provided with the opportunity [57, 68]. Quantitative data to support the view that moving on impacted perseverance was equally limited. Four dropouts were attributed to participants either moving onto other therapy, or dropping out owing to good progress [30, 38, 50, 58, 59, 63, 67].

## Discussion

The findings of this systematic review highlight that perseverance with practice after stroke and factors that influence perseverance have rarely been considered. However, from these findings we have proposed three stages of perseverance (e.g. getting in the game, sticking with it and continuing or moving on). The role that technology plays to support stroke survivors to move through these stages is of particular interest to this review, therefore we discuss conditions required for perseverance within each stage (e.g. acceptability, usability) and how these conditions are mediated by both intrinsic and extrinsic factors (e.g. age, device characteristics, challenge). Further investigation is required to expand our understanding of perseverance with practice for stroke survivors along the rehabilitation and recovery continuum.

Perseverance with stroke rehabilitation was not conceptualised in any studies within this review and no studies contained direct measures of perseverance. Outside of the field of stroke recovery, perseverance has been measured using other scales [69–71] such as the Grit Scale [72] and the Resilience Scale [73]. Unfortunately, none of these measures have been validated for use with stroke survivors [69–73]. The challenge in measuring perseverance is that it is a dynamic behaviour that is mediated day-to-day by intrinsic and extrinsic factors [13, 14] which are yet to be fully understood in the context of stroke rehabilitation.

The proposed framework (Fig. 2) models perseverance into a multi-level concept, which goes some way to improving understanding of the phenomenon. The framework allows clinicians and researchers to identify a stroke survivor’s stage of perseverance, the conditions which are most likely to influence perseverance at that stage, and the key factors which mediate perseverance within each condition. This offers opportunities for clinicians and researchers to develop strategies that target modifiable mediating factors to enhance stroke survivors’ ability to persevere with technology-facilitated home-based UL practice. Strategies to enhance perseverance could be implemented in technology development and clinical prescription of practice.

Further investigation of the proposed framework for perseverance is required. Recognising that perseverance is a dynamic behaviour [13, 14], it will be critical to next use this framework to explore stroke survivors’ perspectives on perseverance in the context of stroke rehabilitation, the factors that mediate perservence, and the relative contribution of each mediating factor to their ability to persevere. Taken together, such information will be critical to informing the development of a stroke specific measure of perseverance, and allow us to design and implement strategies to enhance perseverance.

The findings of this review should be interpreted with caution as none of the studies included specifically set out to explore perseverance with technology-facilitated home-based UL rehabilitation post-stroke. Furthermore, surrogate measures of perseverance (e.g. adherence) were used in the absence of direct measures of perseverance, and while they provided some valuable information, this is insufficient to measure a complex phenomenon such as perseverance [74]. Factors perceived to influence perseverance were identified but supporting quantitative data was extremely limited. Meta-analysis was not possible due to poor adherence reporting and heterogeneity of data that was extracted on stroke survivor cohorts, interventions, and outcome measures. It is possible that some studies may have been missed in the original search or unintentionally excluded. However, authors were systematic in their search and screening process, with two reviewers completing these elements to minimise error and bias. Only four studies within this review achieved a sample size of more than 30 stroke survivors. Lastly, we chose not to exclude poor quality studies based on MMAT score to allow for interpretation of the findings with transparency of quality.

## Conclusion

Technology-facilitated UL rehabilitation offers stroke survivors opportunities to exploit their potential for recovery that will only be realised if they are able to persevere with practice. This review highlighted that the role of perseverance in stroke rehabilitation is yet to be purposefully investigated. We have proposed a framework to conceptualise perseverance with technology-facilitated home-based UL practice which can be used by health professionals to inform prescription of technology for home-based rehabilitation. Ultimately, a stroke survivor’s ability to persevere with technology-facilitated home-based upper limb practice hinges on the acceptability and usability of the technology, and their ability to stay engaged and integrate practice into their everyday life. However, future research into perseverance in the context of stroke rehabilitation is required for ongoing refinement of the framework, and development of a stroke specific measure of perseverance.

## Supplementary Information

**Additional file 1:** Medline search strategy.

**Additional file 2:** Characteristics of upper limb disability.

**Additional file 3:** Characteristics of interventions.

## Data Availability

All data generated or analysed during this study are included in this published article and its supplementary information files.

## Abbreviations

BN: Bridee Neibling
HEP: Home exercise programs
JCU: James Cook University
KH: Kathryn Hayward
MCID: Minimal clinically important difference
MMAT: Mixed methods appraisal tool
PRISMA: Preferred reporting items for systematic reviews and meta-analyses
RB: Ruth Barker
SJ: Sarah Jackson
UL: Upper limb

## Footnotes

1 NVivo 11, QSR International, Melbourne, Australia.

2 Microsoft Excel, Microsoft Corporation, United States of America.
