# Supplementary material for: Perseverance with technology-facilitated home-based upper limb practice after stroke: a systematic mixed studies review
Source: J Neuroeng Rehabil. 2021 Feb 24;18:43. doi: 10.1186/s12984-021-00819-1 (PMC7905577; doi:10.1186/s12984-021-00819-1)
Supplement: Supplementary file 1 — Additional file 1: Medline search strategy. [file 12984_2021_819_MOESM1_ESM.docx]

**APPENDIX 1.** Medline Search Strategy

**Database: MEDLINE**

The search of MEDLINE database was optimised by using the terms developed in consultation with an experienced librarian and the research team.

**Topic: Stroke**

**1.** exp stroke/ or exp "intracranial embolism and thrombosis"/ or exp intracranial hemorrhages/ or exp intracranial arteriovenous malformations/ or exp intracranial aneurysm/

**2.** ("stroke" OR "strokes" OR "cerebrovascular accident" OR "cerebrovascular accidents" OR "cva" OR "cvas" OR "cerebral vascular accident" OR "cerebral vascular accidents" OR "intracranial hemorrhage" OR "intracranial haemorrhage" OR "intracranial hemorrhages" OR "intracranial haemorrhages" OR "intracranial arteriovenous malformation" OR "intracranial arteriovenous malformations" OR "avm" OR "avms" OR "intracranial embolism" OR "intracranial emboli" OR "intracranial thrombosis" OR "intracranial thromboses" OR "intracranial aneurysm" OR "intracranial aneurysms").mp

**Topic: Home**

**3.** exp home care services/

**4.** ("domiciliary" OR "domicile" OR "home" OR "homes" OR "dwelling" OR "dwellings" OR "in-home").mp

**Topic: Rehabilitation**

**5.** exp rehabilitation/ or exp exercise/

**6.** ("habilitation" OR "rehabilitation" OR "neurorehabilitation" OR "therapeutic" OR "therapeutics" OR "therapies" OR "therapy" OR "treatment" OR "treatments" OR "practice" OR "practise" OR "exercise" OR "exercises" OR "physical activities" OR "physical activity" OR "occupational therapy" OR "occupational therapist" OR "physical therapy" OR "physical therapist" OR "physiotherapy" OR "physiotherapist" OR "train" OR "training").mp

**Topic: Technology**

**7.** exp robotics/ or exp self-help devices/ or exp technology/ or exp electronics/ or exp video games/ or exp user-computer interface/ or exp mobile applications/ or exp smartphone/ or exp computers, handheld/ or exp fitness trackers/ or exp virtual reality/ OR exp exoskeleton device/ or exp wearable electronic devices/

**8.** ("remote operation robotics" OR "remote operations robotics" OR "robotics" OR "robotic" OR "soft robotic" OR "soft robotics" OR "telerobotics" OR "robot" OR "robots" OR "assistive device" OR "assistive devices" OR "assistive technologies" OR "assistive technology" OR "self help devices" OR "self-help device" OR "self-help devices" OR "electronic" OR "electronics" OR "computer game" OR "computer games" OR "video game" OR "video games" OR "educational virtual realities" OR "educational virtual reality" OR "instructional virtual realities" OR "instructional virtual reality" OR "virtual reality" OR "augmented reality" OR "augmented realities" OR "educational augmented realities" OR "educational augmented reality" OR "instructional augmented realities" OR "instructional augmented reality" OR "user computer interface" OR "user computer interfaces" OR "user-computer interface" OR "user-computer interfaces" OR "virtual system" OR "virtual systems" OR "mobile app" OR "mobile application" OR "mobile applications" OR "mobile apps" OR "portable electronic app" OR "portable electronic application" OR "portable electronic applications" OR "portable electronic apps" OR "portable software app" OR "portable software application" OR "portable software applications" OR "portable software apps" OR "handheld computer" OR "handheld computers" OR "pda computer" OR "pda computers" OR "palm pilot" OR "palm pilots" OR "palm-top computer" OR "palm-top computers" OR "palmtop computer" OR "palmtop computers" OR "personal digital assistant" OR "pocket pc" OR "pocket pcs" OR "tablet computer" OR "tablet computers" OR "smart phone" OR "smart phones" OR "smartphone" OR "smartphones" OR "activity tracker" OR "activity trackers" OR "fitness tracker" OR "fitness trackers" OR "personal fitness tracker" OR "personal fitness trackers" OR "physical fitness tracker" OR "physical fitness trackers" OR "electronic skin" OR "wearable device" OR "wearable devices" OR "wearable electronic device" OR "wearable sensor" OR "wearable sensors" OR "wearable electronic devices" OR "wearable technologies" OR "wearable technology" OR "exoskeleton device" OR "exoskeleton devices" OR "robotic exoskeleton" OR "robotic exoskeletons" OR "internet of thing" OR "internet of things" OR "internet-of-thing" or "internet-of-things" OR "fitbit" OR "garmin" OR "misfit" OR "xiaomi" OR "moov" OR "samsung" OR "huawei" OR "tomtom" OR "amazfit" OR "polar" OR "motiv" OR "apple" OR "gamification" OR "cell phone" OR "cell phones" OR "cellphone" OR "cellphones" OR "cellular phone" OR "cellular telephone" OR "mobile telephone" OR "mobile phone" OR "mobile phones").mp

**Topic: Upper Limb**

**9.** exp Upper Extremity/

**10.** ("upper extremities" OR "upper extremity" OR "upper limb" OR "upper limbs" OR "arm" OR "arms" OR "upper arm" OR "upper arms" OR "elbow" OR "forearm" OR "forearms" OR "hand" OR "hands" OR "finger" OR "fingers" OR "wrist" OR "wrists" OR "shoulder" OR "shoulders").mp

**Combine**

**11.** 1 OR 2

**12.** 3 OR 4

**13.** 5 OR 6

**14.** 7 OR 8

**15.** 9 OR 10

**16.** 11 AND 12 AND 13 AND 14 AND 15

This search was customised for the other databases (CINAHL, PsychINFO, Scoups, Web of Science, EmCare).
