# Supplementary material for: Perseverance with technology-facilitated home-based upper limb practice after stroke: a systematic mixed studies review
Source: J Neuroeng Rehabil. 2021 Feb 24;18:43. doi: 10.1186/s12984-021-00819-1 (PMC7905577; doi:10.1186/s12984-021-00819-1)
Supplement: Supplementary file 2 — Additional file 2: Characteristics of upper limb disability. [file 12984_2021_819_MOESM2_ESM.docx]

**ADDITIONAL FILE 2.** Characteristics of Upper Limb Disability

| **Author (Year)** | **Stroke Survivor Upper Limb Disability Inclusion Criteria** |
| --- | --- |
| Adie (2017) [28] | UL weakness defined as MRC Scale power less than 5 in any joint plane and able to manipulate the Wii™ remote control |
| Alankus (2010) [29] | UL disability inclusion criteria not described in this study |
| Basteris (2015) [30] | Partial central paresis of the UL and hand with 15° active elbow flexion and a quarter range of active finger flexion |
| Bernocchi (2018) [31] | mAS ≤ 3 |
| Bhattacharjya (2015) [32] | UL disability inclusion criteria not described in this study |
| Brokaw (2015) [33] | Right hemiplegia. FMA 35, SIS-16 67, unable to complete any tasks on JHFT. |
| Buick (2016) [34] | some voluntary control of shoulder/elbow movement, and room for improvement (maximum score of 40/57) on the ARAT |
| Burdea (2019) [35] | UL unilateral motor involvement (FMA score 10 to 50); ability to actively move UL more than 15° for shoulder and elbow flexion/extension |
| Butler (2014) [36] | Limited activities of daily living with affected hand consistent with a score of 1-3 on the motor UL item of the NIHSS |
| Donoso-Brown (2014) [37] | Impairment ranged from no active extension in the digits to full digit extension |
| Donoso-Brown (2015) [10] | Unilateral hemiparesis and motor impairments ranging from difficulty with handling objects to no active hand movement |
| Emmerson (2017) [38] | Any degree of impairment to UL function |
| Emmerson (2018) [39] | Any degree of impairment to UL function |
| Fluet (2019) [40] | FMA UL of 36-58/66 |
| Fu (2019) [41] | FMA UL Score ≤ 11 |
| Hayward (2015) [42] | Triceps MMT <3/5 and MAS (Item 6) Score <4 |
| Housley (2016) [43] | NIHSS of 1–3; Some degree of UL voluntary activity, as indicated by the ability to move their proximal and/or distal joints against gravity |
| Jordan (2014) [44] | MRC strength grade 2-4 for shoulder and elbow flexion |
| King (2012) [45] | Limited voluntary movement in their affected UL |
| Langan (2013) [46] | UL disability inclusion criteria not described in this study |
| Lin (2013) [47] | UL hemiparesis affecting the right or left UL |
| Linder (2013) [48] | UL disability inclusion criteria not described in this study |
| Linder (2015) [49] | FMA score of 11–55 |
| Nijenhuis (2017) [51] | Movement limitations in the UL, but ≥15° active flexion of the elbow and active flexion of the finger(s) ≥25% of the passive range of motion |
| Nijenhuis (2015) [50] | Partial central paresis of the UL with 15° active elbow flexion and a quarter range of active finger flexion |
| O-Brien Cherry (2017) [52] | Persistent hemiparesis as indicated by a score of 1–3 on the motor UL item of the NIHSS |
| Pareto (2011) [53] | Box and blocks score <45 |
| Parker (2014) [54] | UL disability inclusion criteria not defined but described as average AROM Shoulder Flexion 60 degrees (range 20-90 degrees), average AROM Shoulder Abduction 49 degrees (range 20-90 degrees) |
| Proffitt (2011) [55] | UL disability inclusion criteria not described in this study |
| Proffitt (2015) [56] | UL disability inclusion criteria not described in this study |
| Rand (2015) [57] | FMA UL sub-score ranging from 15 to 55 |
| Sivan (2014) [58] | Residual weakness of UL and a minimum of some voluntary UL movement to perform the hCAAR exercise tasks including suitable hand grip function. In sitting position must be able to actively move affected hand, rested on table, by at least 15cm. |
| Sivan (2016) [59] | Residual weakness of UL and a minimum of some voluntary UL movement to perform the hCAAR exercise tasks including suitable hand grip function. In sitting position must be able to actively move affected hand, rested on table, by at least 15cm. |
| Slijper (2014) [60] | NIHSS <15 |
| Standen (2015) [61] | Residual UL dysfunction |
| Standen (2017) [62] | Residual UL impairment |
| Szturm (2020) [63] | Actively extend at least 10 degrees at the metacarpophalangeal and interphalangeal joints, extend 10 degrees at the wrist, and had at least 30 degrees of active flexion-extension of the elbow and shoulder |
| Thielbar (2020) [64] | Moderate arm impairment (stage 3-5 on Stage of Arm of Chedoke-McMaster Stroke Assessment) |
| Wingham (2015) [65] | Able to independently manipulate the Wii™ remote control |
| Wittmann (2016) [66] | UL hemiparesis, the ability to lift the paretic UL against gravity, a minimal UL workspace of 20 cm x 20 cm in the horizontal plane |
| Wolf (2015) [67] | FMA score of 11-55 |
| Yacoby (2019) [68] | Mild to moderate UL weakness – defined by a FMA UL subtest score between 15 and 55 |

**ARAT –** Action Research Arm Test; **AROM**  - Active Range of Motion; **FMA –** Fugl-Meyer Assessment; **hCAAR** – Home-based Computer Assisted Arm Rehabilitation; **JHFT** – Jebsen Hand Function Test; **MAS** – Motor Assessment Scale; **mAS** – Modified Ashworth Scale; **MMT** – Manual Muscle Test; **MRC** – Medical Research Council; **NIHSS –** National Institutes of Health Stroke Scale; **SIS-16 –** Stroke Impact Scale (16 Item); **UL** – Upper Limb
