# Supplementary material for: Perseverance with technology-facilitated home-based upper limb practice after stroke: a systematic mixed studies review
Source: J Neuroeng Rehabil. 2021 Feb 24;18:43. doi: 10.1186/s12984-021-00819-1 (PMC7905577; doi:10.1186/s12984-021-00819-1)
Supplement: Supplementary file 3 — Additional file 3: Characteristics of interventions. [file 12984_2021_819_MOESM3_ESM.docx]

**ADDITIONAL FILE 3.** Characteristics of Interventions

| **Author (Year)** | **Technology** | | | | | | | | |
| --- | --- | --- | --- | --- | --- | --- | --- | --- | --- |
|  | **Therapy Tool** | **Classification** | **Hardware and Software** | **CA**  **(Y/N)** | **RS**  **(Y/N)** | **Set-up Assist** | **Device Assist** | **Feedback** | **Progression** |
| Adie (2017) [28] | Nintendo Wii Sports ™ | Sensor, Motion based games | Hardware: Nintendo Wii™ Console and Remotes Software: Wii Sports Video Game | Y | N | NR | None | Technology – during and post-training Therapist – phone call once/week | A(PB), M |
| Alankus (2010) [29] | Looking Glass | Sensor, Motion based games | Hardware: Laptop computer, 2 x Wii™ remotes, webcam Software: Motion based games | N | Y | Mixed – Independent or therapist/carer /family member | None | Technology – during and post-training Therapist – clinic visit once/week Participant – self feedback on own goals | A(MI), M |
| Basteris (2015) [30] | SCRIPT | Sensor, Motion based games | Hardware: Passive-actuated hand and wrist orthosis, computer (touchscreen display), Saebo™ mobile arm support Software: Motion based gamess, telesupervision platform | N | Y | NR | Partial | Technology – during and post-game Therapist – phone calls (3) in week one; home visit once/week from week two; remote offline monitoring | A(PB), M |
| Bernocchi (2018) [31] | Glorhea Lite™ | Robotic, Sensor, Motion based games | Hardware: Glorhea Lite™ rehabilitation glove, computer  Software: Multisensory (visual, auditory and kinaesthetic) 3D simulation games | Y | Y | Assistance – Carer/family member | Partial-full | Technology – during and post training Therapist – phone call, video-conference, or home visit once/fortnight | A(PB), M |
| Bhattacharjya (2019) [32] | mRehab | Sensor | Hardware: Google Nexus 5 smartphone, 3D-printed functional items (e.g. bowl, mug, doorknob, key) Software: mRehab customised app | N | Y | Independent | None | Technology – during and post-training | M |
| Brokaw (2015) [33] | HAMSTER | Sensor, Motion based games | Hardware: HandSOME passive exoskeleton, computer, Microsoft Kinect™ Software: Motion based games | N | Y | Independent | Partial | Technology – during and post training Therapist – three training sessions (clinic x 1, home x 2), phone calls x 7 days following Participant – phone calls x 2 | A(PB) |
| Buick (2016) [34] | ReJoyce | Sensor, Motion based games | Hardware: ReJoyce workstation, laptop, webcam, FES wrist cuff, toothclick detecting earpiece  Software: Motion based games, telerehbilitation | Y | Y | Independent | None | Technology – during and post-training Therapist – during sessions via telerehabilitation | A(PB), M |
| Burdea (2019) [35] | BirghtBrainer | Sensor, Motion based games | Hardware: BrightBrainer System, BrightBrainer Grasp controller, HTC VIVE™ controller, 60”x30” table covered with X-ray Pad.  Software: Motion based games | N | Y | NR | Partial | Technology – during and post-training | A(PB) |
| Butler (2014) [36] | HandMentor Pro™ | Robotic, Sensor, Motion based games | Hardware: computer control box, pneumatic arm unit and data collection device, and communications module Software: Motion based games | Y | Y | Assistance – Carer/family member | Partial-full | Technology – during and post-training Therapist – daily remote offline monitoring, phone call once/week | A(PB), M |
| Donoso-Brown (2014) [37] | NGT | Sensor, Motion based games | Hardware: Laptop computer, NGT console, sEMG leads and electrodes, internet connection Software: Peggle™ game | N | Y | Mixed – Independent or carer/family member | None | Technology – during and post-training Therapist – phone call once/week | A(PB), M |
| Donoso-Brown (2015) [10] | NGT | Sensor, Motion based games | Hardware: Laptop computer, NGT console, sEMG leads and electrodes, internet connection Software: Peggle™ game | N | Y | Mixed – Independent or carer/family member | None | Technology – during and post-training Therapist – phone call once/week | A(PB), M |
| Emmerson (2017) [38] | iPad | Tablet | Hardware: iPAD Software: video recording, reminder alarm | Y | N | Mixed – Independent or carer/family member | None | Technology – during training  Therapist – clinic visit (variable timing) | M |
| Emmerson (2018) [39] | iPad | Tablet | Hardware: iPAD Software: video recording, reminder alarm | Y | N | Mixed – Independent or carer/family member | None | Technology – during training  Therapist – clinic visit (variable timing) | M |
| Fluet (2019) [40] | HoVRS | Sensor, Motion based games | Hardware: Leap Motion controller, passive arm support (Armon™ Edero or Saebo™ Mobile Arm Support) Software: Motion based games | N | Y | Independent | Partial | Technology – during and post training | A(PB) |
| Fu (2019) [41] | CCFES | Sensor, Motion based games | Hardware: Computer, bend sensor, fingerless mitten, CCFES device Software: Motion based games | N | Y | Mixed – Independent or carer/family member | Partial-full | Technology – during and post-training Therapist – clinic visit twice/week | M |
| Hayward (2015) [42] | SMART Arm | Sensor | Hardware: SMART Arm device (computer, FES, frame) Software: Motion display | N | Y | Assistance – Carer/family member | Partial-full | Technology – during and post-training Therapist – home visits x 2, phone calls x 6 | A(PB), M |
| Housley (2016) [43] | HandMentor Pro™ | Robotic, Sensor, Motion based games | Hardware: computer control box, pneumatic arm unit and data collection device, and communications module Software: Motion based games | Y | Y | Mixed – Independent or carer/family member | Partial-full | Technology – during and post-training Therapist – phone call once/week, daily remote offline monitoring | A(PB), M |
| Jordan (2014) [44] | Smart Skate | Sensor, Motion based games | Hardware: Smart Skate Device (skateboard, computer, mouse) Software: Motion based games | Y | Y | Assistance – Therapist | Partial | Technology – during and post-training Therapist – home visit all sessions | A(PB), M |
| King (2012) [45] | CyWee Z | Sensor, Motion based games | Hardware: CyWee Z controller, computer  Software: Motion based games | Y | Y | Independent | None | Technology – during and post-training | A(PB) |
| Langan (2013) [46] | Stereognosis Training System | Sensor | Hardware: Laptop, equipment for modular tasks, sensor target board Software: Motion and object display | N | Y | NR | None | Therapist – video-conference daily initially and decreasing to once/week in the final week Participant – during stereognosis tasks | NR |
| Lin (2013) [47] | Looking Glass | Sensor, Motion based games | Hardware: Computer, Wii™ remotes, webcam Software: Motion based games | Y | Y | NR | None | Technology – during and post-training | A(MI), M |
| Linder (2013) [48] | HandMentor Pro™ | Robotic, Sensor, Motion based games | Hardware: computer control box, pneumatic arm unit and data collection device, and communications module Software: Motion based games | Y | Y | NR | Partial-full | Technology – during and post-training Therapist – phone call once/week | A(PB), M |
| Linder (2015) [49] | HandMentor Pro™ | Robotic, Sensor, Motion based games | Hardware: computer control box, pneumatic arm unit and data collection device, and communications module Software: Motion based games | Y | Y | Assistance – Carer/family member | Partial-full | Technology – during and post-training Therapist – phone call once/week | A(PB), M |
| Nijenhuis (2015) [50] | SCRIPT | Sensor, Motion based games | Hardware: Passive-actuated hand and wrist orthosis, computer (touchscreen display), Saebo™ mobile arm support Software: Motion based games, telesupervision platform | N | Y | NR | Partial | Technology – during and post-training Therapist – home visit once/week, remote offline monitoring | A(PB), M |
| Nijenhuis (2017) [51] | SCRIPT | Sensor, Motion based games | Hardware: Passive-actuated hand and wrist orthosis, computer (touchscreen display), Saebo™ mobile arm support Software: Motion based games, telesupervision platform | N | Y | NR | Partial | Technology – during and post-training Therapist – home visit once/week, remote offline monitoring | A(PB), M |
| O-Brien Cherry (2017) [52] | HandMentor Pro™ | Robotic, Sensor, Motion based games | Hardware: computer control box, pneumatic arm unit and data collection device, and communications module Software: Motion based games | Y | Y | Assistance – Carer/family member | Partial-full | Technology – during and post-training | A(PB), M |
| Pareto (2011) [53] | Curictus Immersive Workbench | Sensor, Motion based games | Hardware: Game based immersive workbench, haptic stick, video-conferencing system, computer, webcam, headset Software: web based patient care management system, motion based games | N | Y | Supervision – Therapist | None | Technology – during and post-training Therapist – video-conference all sessions | NR |
| Parker (2014) [54] | SMART System | Sensor | Hardware: Computer, inertial trackers x 3 (upper arm, wrist and chest) Software: Motion display | N | Y | NR | None | Technology – during and post-training | NR |
| Proffitt (2011) [55] | Looking Glass | Sensor, Motion based games | Hardware: Computer, Wii™ remotes, webcam Software: Motion based games | Y | Y | NR | None | Technology – during and post-training Therapist – clinic visit once/week | A(MI), M |
| Proffitt (2015) [56] | Mystic Isle | Sensor, Motion based games | Hardware: Computer, Microsoft Kinect™ Sensor, wireless mouse Software: Motion based games | Y | Y | Mixed – Independent or carer/family member | None | Technology – during and post-training | A(PB), M |
| Rand (2015) [57] | Microsoft X-box Kinect™ or Playstation 2 Eyetoy™ | Sensor, Motion based games | Hardware: Microsoft X-box Kinect™, Playstation 2 Eyetoy™ Software: Motion based games | Y | N | Mixed – Independent or therapist/carer /family member | None | Technology – during and post-training Therapist – phone call once/day (week 1) and once/week (week 2-5), home visit x1 (week 2) | A(PB) |
| Sivan (2014) [58] | hCAAR | Sensor, Motion based games | Hardware: Computer, joystick on chassis Software: Motion based games | N | Y | Mixed – Independent or carer/family member | Partial | Technology – during and post-training | A(PB), M |
| Sivan (2016) [59] | hCAAR | Sensor, Motion based games | Hardware: Computer, joystick on chassis Software: Motion based games | N | Y | Mixed – Independent or carer/family member | Partial | Technology – during and post-training | A(PB), M |
| Slijper (2014) [60] | Gaming Console | Sensor, Motion based games | Hardware: Laptop computer embedded into a gaming console, 2 x handles attached to console with string Software: Motion based games | N | Y | NR | None | Technology – during and post-training Therapist – clinic visit once/week | A(PB) |
| Standen (2015) [61] | Virtual Glove | Sensor, Motion based games | Hardware: Hand mounted power unit with LEDs, Nintendo Wii™ Remotes, Computer Software: Motion based games | N | Y | Mixed – Independent or therapist/carer /family member | None | Technology – during and post-training Therapist – home visit once/week or fortnight | A(PB), M |
| Standen (2017) [62] | Virtual Glove | Sensor, Motion based games | Hardware: Hand mounted power unit with LEDs, Nintendo Wii™ Remotes, Computer Software: Motion based games | N | Y | Mixed – Independent or therapist/carer /family member | None | Technology – during and post-training Therapist – home visit once/week or fortnight | A(PB), M |
| Szturm (2020) [63] | GTP | Sensor, Motion based games | Hardware: Laptop, inertial based computer mouse Software: Motion based games (www.bigfishgames.com) | N | Y | NR | None | Technology – during training Therapist – clinic visit (3 x initial training sessions then once/fortnight), phone call or email once/week | A(PB), M |
| Thielbar (2020) [64] | VERGE | Sensor, Motion based games | Hardware: Microsoft Kinect™, computer, wireless mouse Software: Motion based games | N | Y | NR | None | Technology – during training | NR |
| Wingham (2015) [65] | Nintendo Wii Sports ™ | Sensor, Motion based games | Hardware: Nintendo Wii™ Console and Remotes  Software: Wii Sports Video Game | Y | N | Mixed – Independent or carer/family member | None | Technology – during and post-training Therapist – phone call once/week | A(PB), M |
| Wittmann (2016) [66] | ArmeoSenso System | Sensor, Motion based games | Hardware: Laptop computer, inertial trackers x 3 (upper arm, lower arm and trunk) Software: Motion based games | N | Y | NR | None | Technology – during and post-training | A(PB) |
| Wolf (2015) [67] | HandMentor Pro™ | Robotic, Sensor, Motion based games | Hardware: computer control box, pneumatic arm unit and data collection device, and communications module Software: Motion based games | Y | Y | Assistance – Carer/family member | Partial-full | Technology – during and post-training Therapist – phone call once/week | A(PB), M |
| Yacoby (2019) [68] | Microsoft X-box Kinect™ or Playstation 2 Eyetoy™ | Sensor, Motion based games | Hardware: Microsoft X-box Kinect™, Playstation 2 Eyetoy™ Software: Motion based games | Y | N | Mixed – Independent or therapist/carer /family member | None | Technology – during and post-training Therapist – phone call once/day (week 1) and once/week (week 2-5), home visit x1 (week 2) | A(PB) |

**AMES –** Assisted Movement with Enhanced Sensation; **A(MI)** – Automatic (Machine Initiated); **A(PB) –** Automatic (Performance Based); **CA –** Commercially Available; **CCFES –** Contralaterally Controlled Functional Electrical Stimulation; **FES –** Functional Electrical Stimulation; **GTP –** Game-assisted Telerehabilitation Platform; **HAMSTER –** Home Arm Movement Stroke Training Environment; **HandSOME** – Hand Spring Operated Movement Enhancer; **hCARR –** Home-based Computer Assisted Arm Rehabilitation; **HoVRS –** Home Virtual Rehabilitation System; **LEDs –** Light Emitting Diodes; **M –** Manual; **mRehab –** Mobile Rehabilitation; **N –** No; **NGT –** Neurogame Therapy; **NR –** Not Reported; **ReJoyce –** The Rehabilitation Joystick for Arm and Hand Exercises; **RS –** Rehabilitation Specific; **SCRIPT –** Supervised Care and Rehabilitation Involving Personal Telerobotics; **sEMG –** Surface Electromyographic; **SMART Arm –** Sensorimotor Active Rehabilitation Training of the Arm; **VERGE –** Virtual Environment for Rehabilitative Gaming Exercises; **Y –** Yes
